# Imaging assessment of penetrating injury of the neck and face

**DOI:** 10.1007/s13244-012-0191-y

**Published:** 2012-09-04

**Authors:** Curtis Offiah, Edward Hall

**Affiliations:** Department of Radiology, The Royal London Hospital, Barts Health NHS Trust, Whitechapel London, E1 1BB UK

**Keywords:** MDCTA, Penetrating injury, Face, Neck, Bullet, Knife

## Abstract

**Background:**

Penetrating trauma of the neck and face is a frequent presentation to acute emergency, trauma and critical care units. There remains a steady incidence of both gunshot penetrating injury to the neck and face as well as non-missile penetrating injury—largely, but not solely, knife-related. Optimal imaging assessment of such injuries therefore remains an on-going requirement of the general and specialised radiologist.

**Methods:**

The anatomy of the neck and face—in particular, vascular, pharyngo-oesophageal, laryngo-tracheal and neural anatomy—demands a more specialised and selective management plan which incorporates specific imaging techniques.

**Results:**

The current treatment protocol of injuries of the neck and face has seen a radical shift away from expectant surgical exploration in the management of such injuries, largely as a result of advances in the diagnostic capabilities of multi-detector computed tomography angiography (MDCTA), which is now the first-line imaging modality of choice in such cases.

**Conclusion:**

This review aims to highlight ballistic considerations, differing imaging modalities, including MDCTA, that might be utilised to assist in the accurate assessment of these injuries as well as the specific radiological features and patterns of specific organ-system injuries that should be considered and communicated to surgical and critical care teams.

***Teaching points*:**

*• MDCTA is the first-line imaging modality in penetrating trauma of the neck and, often, of the face*

*• The inherent deformability of a bullet is a significant factor in its tissue-damaging capabilities*

*• MDCTA can provide accurate assessment of visceral injury of the neck as well as vascular injury*

*• Penetrating facial trauma warrants radiological assessment of key adjacent anatomical structures*

*• In-driven fragments of native bone potentiate tissue damage in projectile penetrating facial trauma*

## Introduction

Penetrating injuries of the neck and face as a result of projectile (gun) and non-projectile (knives and other sharp implements such as screwdrivers or glass) mechanisms represent a significant source of acute admission of civilians to Accident and Emergency Departments and Trauma Units in the United Kingdom. This trend is reflected across the rest of Europe. Although accidental blunt trauma to the neck is a commoner cause of acute trauma presentation, national statistics for 2009/10 confirm a general progressive rise in the number of homicides involving knives and sharp instruments over the last five decades [1, 2]. In the United Kingdom, the number of actual bodily harm and grievous bodily harm offences involving a knife or sharp instrument have remained more or less constant between 2009 and 2010, accounting for 4 % of violent and sexual offences recorded by the police [1, 2]. Fatalities caused by a firearm in the United Kingdom (statistics for England and Wales) also remained constant between 2009 and 2010; however, serious injuries increased by 3 % between 2008/2009 and 2009/2010, with firearms offences geographically concentrated in the three most heavily populated police force areas and accounting for two-thirds of all such offences (recorded by the police). [1, 2].

The radiological evaluation of penetrating neck and face injuries can be daunting given the emergency circumstances requiring imaging; in addition, given that this particular anatomical area of the body combines vascular, gastrointestinal, respiratory, endocrine, lymphatic, skeletal and nervous systems, imaging of penetrating neck injuries can be challenging.

## Location and classification

**Fig. 1:**
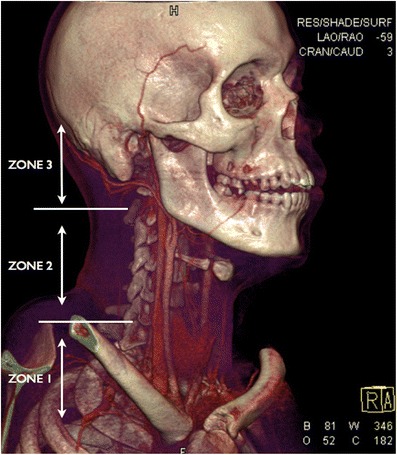
Three-dimensional volume rendering technique (3D VRT) of MDCTA performed on a young male victim of a stab wound to the neck demonstrating the anatomical trauma zones in penetrating injury of the neck. In this young patient, the cricoid cartilage has not yet calcified and therefore is not visualised on the VRT but the thyroid gland is visible (the lower border of the cricoid cartilage will be located just above the level of the isthmus of the thyroid gland)

**Fig. 2:**
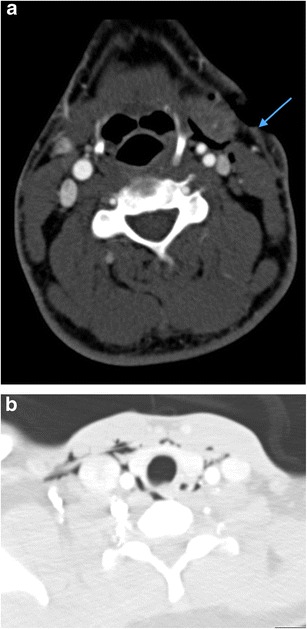
**a** Axial image of MDCTA performed on a 33-year-old man who sustained a stab injury to left zone 2. The platysma has been breached (*arrow*) and there is soft tissue emphysema in the deep cervical fascia surrounding the left carotid sheath. No major vascular injury or upper aerodigestive tract penetration was present. There was no pneumothorax. **b** Axial image of MDCTA through zone 1 on lung-window settings of a different patient who sustained left zone 2 penetrating knife injury with breach of platysma (image not shown). Diffuse soft tissue emphysema is noted in the deep cervical fascia bilaterally in zone 1 despite penetrating wound entry in left zone 2. This can be a common finding in penetrating neck injury where platysma has been breached even in the absence of perforation of the upper aerodigestive tract or pneumothorax. Nevertheless, penetration of the laryngopharynx should be further excluded where clinical and radiological suspicion is high. (Contrast swallow was negative in this patient)

Historically, by the end of World War II, the unacceptably high mortality rate related to penetrating neck wounds (because of associated neurovascular injury going unrecognised) prompted a shift in the management of such wounds towards mandatory exploration of all surgically accessible injuries that penetrated the platysma [3–7]. It was much later that Monson et al. and Roon and Christiensen [3, 7, 8] attempted to standardise reporting of penetrating neck injuries and improve outcomes by dividing the neck into three anatomic zones of injury. These three anatomical zones are described as follows: zone 1 extends from the level of the clavicles and sternal notch to the cricoid cartilage; zone 2, the largest and most exposed area, extends from the level of the cricoid cartilage to the angle of the mandible; zone 3 extends from the level of the angle of the mandible to the base of the skull (Fig. 1). Traditionally, zone 1 and zone 3 injuries were largely managed conservatively because of difficulties relating to surgical access, with surgery reserved for very few, selected cases . Studies of civilian experience of penetrating neck injury in the latter part of the twentieth century by groups such as Monson et al. revealed that the mandatory exploration of the surgically accessible zone 2 yielded high rates of negative explorations [3, 9–21]. This has led to a current modern-day management paradigm of selective neck exploration based on clinical and radiological criteria. The emergence of multi-detector computed tomography (MDCT) technology has facilitated the use of CT angiography (CTA) as a reliable, sensitive, fast and non-invasive radiological imaging modality in the assessment of penetrating neck injuries [18, 19, 21–24] (Fig. 2a, b). CTA has allowed detection of direct and indirect signs of vascular injury not only in zone 2 but also in zone 1 and zone 3; it has also allowed valuable radiological assessment of non-vascular structures in the neck such as the upper aerodigestive tract and cervical spine. CTA has been shown to significantly reduce the number of negative surgical neck explorations [3, 5, 18, 19, 22–26].

**Fig. 3:**
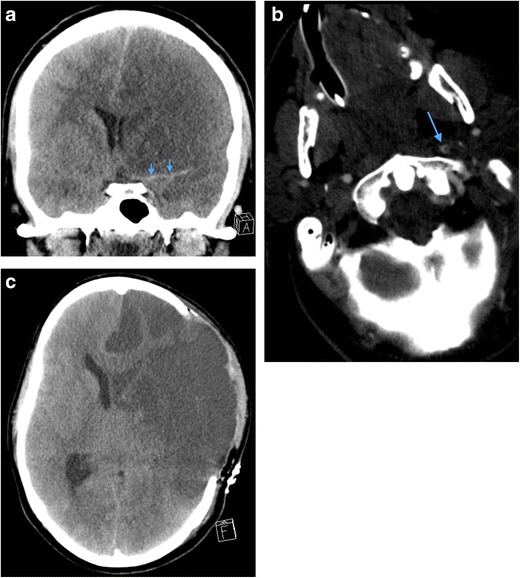
**a** Coronal reformatted non-contrast CT brain image of a 34-year-old man who sustained a penetrating knife injury to the left zone 2 of the neck requiring emergency surgical neck exploration prior to imaging assessment because of catastrophic haemorrhage. Therefore, pre-treatment CT and MDCTA were not performed because of the clinically evident active life-threatening haemorrhage on arrival in the emergency department. The non-contrast CT brain assessment was performed immediately prior to the post-operative MDCTA (**b**) and demonstrates acute thromboembolic “malignant” left anterior circulation infarction: there is already ischaemic demarcation of both the left middle and anterior cerebral artery territories with severe subfalcine herniation and midline shift; hyperdense thromboembolus is evident in the left middle cerebral artery (*arrows*) and proximal left anterior cerebral artery. **b** Axial image of MDCTA of the same patient as in **a** performed immediately post-operatively following internal carotid artery repair reveals a tail of extensive intraluminal thrombus extending distally from the site of vessel injury and repair (*arrow*). **c** Axial non-contrast CT brain of the same patient as in **a** and **b** performed 48 h later. The patient has undergone interval emergency left decompressive hemicraniectomy: the left anterior circulation low attenuation infarction is more established and the significant mass effect, subfalcine herniation and intracranial pressure related to swollen oedematous parenchyma have been ameliorated by hemicraniectomy. Unfortunately, brain-stem death was confirmed 12 h later

With the significant overall reduction in surgical neck exploration in patients sustaining penetrating neck injury, the use of the zonal descriptive classification has fallen out of favour to a degree; improved cross-sectional CT imaging of such injuries also reveals that, despite the identification of an entry wound in one zone, the track of the penetrating injury may traverse other zones. Nevertheless, the authors still favour the use of such descriptive classification to assist the treating trauma surgeon and recommend familiarity with the classification by any reporting radiologist. It is worth noting that a proportion of patients with penetrating neck injury will require immediate surgical intervention without radiological assessment: current indications for such immediate surgical intervention include: those patients with an expanding haematoma, haemorrhagic exsanguination, shock, airway compromise and massive subcutaneous emphysema [27] (Fig. 3a–c). Nevertheless, the number of patients requiring MDCT angiographic assessment for penetrating neck injury may be considerable in any busy trauma unit. The authors audited the number of patients undergoing emergency CT imaging for projectile and non-projectile penetrating trauma of any part of the body in a 12-month period at their institution (a total of 456 cases): the number of CT angiograms undertaken for non-projectile penetrating neck injury in a 12-month period was also evaluated as part of this audit and 93 such patients were identified. Of these, breach of platysma was demonstrated on multi-detector CT angiography (MDCTA) in 72 %.

## Anatomy

Description of the detailed anatomy of the neck is beyond the scope of this review. However, the most salient considerations include the distinctive fascial envelopes in the neck. The neck is invested in two fascial layers: the superficial fascia and the deep cervical fascia. The superficial fascia overlies the platysma and forms part of the superficial musculo-aponeurotic system in the face; the deep cervical fascia is made up of the investing layer, pretracheal layer, prevertebral layer and carotid sheath. Penetrating injuries confined to the superficial fascia—that is, injuries not breaching the platysma—are not life-threatening.

## Pathophysiology of gunshot wounds and ballistics

The mechanisms by which projectiles, such as those discharged from firearms (bullets, for example), cause tissue injury (terminal ballistics) are crushing and stretching—that is, shockwave formation and temporary cavitation [28–30]. Harvey and Korr [30] described two types of tissue damaging pressure waves produced by projectiles such as bullets: the first wave, the sonic shock wave, relates to the sound of the projectile striking the target; it transmits at the speed of sound (faster than the projectile itself) and no temporary cavity is associated with this sonic shock wave; the secondary pressure wave, the temporary cavity, is formed when the penetrating projectile strikes and deforms tissue and radiates out laterally, propagating tissue damage.

**Fig. 4:**
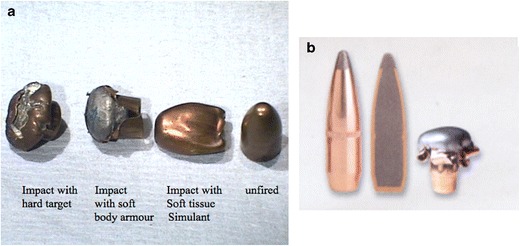
**a** Photograph of full-metal jacket bullets (9-mm pistol bullets) exhibiting the appearances of an unfired bullet and the relative deformation exhibited by discharged bullets which have struck targets of varying density. The copper metal jacket is evident around the lead core in the deformed bullets. **b** Photograph of soft-point hunting bullets depicting the soft-point construct and incomplete hard-metal jacket; significant deformation of this bullet caused by target impact is also depicted

The lethality of a projectile relates significantly and directly to its deformability and the degree of fragmentation it undergoes in the target. The Hague Convention of 1899 has strict specifications for military-use bullets in order to limit the lethality of such ammunition; true full-metal jacketed military bullets resist deformation and fragmentation and are in fact designed to wound but not to kill (Fig. 4a). The harsh reality of warfare philosophy is that the soldier wounded but not killed by a bullet on the frontline is a far greater drain on the economical and manpower resources of the enemy than is a fatality. Clearly, the construction of some projectiles such as Improvised Explosive Devices (IED) previously and currently employed in certain international conflicts by certain factions do not adhere to the Hague Convention and the injury potential of such devices is well recognised. Similarly, civilian (and hunting) ammunition does not adhere to the restrictions of the Convention and is therefore capable of causing tremendous tissue damage [28, 29].

In general, bullet wounds (and, in fact, any projectile wound) are more severe when the projectile yaws through tissue, when the projectile fragments or deforms (into a mushroom shape, for example), when the projectile is large, or when the projectile is travelling at high velocity [28]. One of the “recommendations” of the hollow-point and soft-point bullet (the former are used by specialist fire-arms officers in the UK) is that they are less likely to exit the target, which poses smaller risk to bystanders. This is because they exhibit significant deformation as a result of their construct, transferring most or all of their kinetic energy to the tissue and usually stopping within the body target. The lethality of the hollow-point and soft-point bullet (Fig. 4b) is therefore very high and it is for this reason that this type of ammunition is also used in hunting, supposedly expediting death of the target animal as rapidly and as humanely as possible.

The terminal ballistics of shotgun injuries warrant special consideration: shotgun pellets create tissue damage proportional to the distance from the target, proportional to the number of pellets that hit the target, and proportional to the size and pattern of the pellet strike. At close range, shotguns inflict massive tissue damage and large avulsion wounds. However, this type of weapon is less effective at greater distances from the target, with projectile velocity falling considerably beyond 20–40 m.

## CT technique

MDCT scanning is now widely available and MDCTA is the recommended primary imaging modality and method of choice. This allows not only vascular assessment but also appropriate radiological evaluation of the osseous cervical spine and skull base, of the craniofacial skeleton and valuable assessment of the viscera and fascial planes of the neck and face. At the authors’ institution, both 64- and 128-MDCT imaging (Siemens) is available but all such scans are now currently performed at 128-slice capability. CT protocol parameters currently in use at the authors’ institution are described in brief: a collimation of 128 × 0.6 mm (for the 128-detector scanner) or 64 × 0.6 mm (for the 64-slice scanner); 120 kV is operated on both types of scanner and a reference mAs of 220 on the 64-slice scanner and 120 on the 128-slice scanners; Siemens care kV is utilised on the 128-slice scanners and CareDose4D is utilised on all scanners; 100 ml intravenous contrast is injected for CT angiographic assessment of the neck vessels; where additional CT angiographic assessment of the intracranial vessels is required, a 50 ml normal saline “chaser” is injected following the intravenous contrast injection. A contrast injection rate of 5 ml per second through (at smallest) a 19-G venous cannula is used. Bolus-tracking from the arch of the aorta is routinely operated with a delay of 7 s after triggering of contrast injection (initiated at 100 Houndsfield units) on the 128-slice scanners but a delay of 6 s on the 64-slice scanner.

Liberal use of multiplanar reformats, always in correlation with the axial source dataset images, is advised. Appropriate window-level and window-width settings for the different anatomical regions (for example, the osseous cervical spine and skull base) should also be utilised.

## Extracranial vascular trauma

**Fig. 5:**
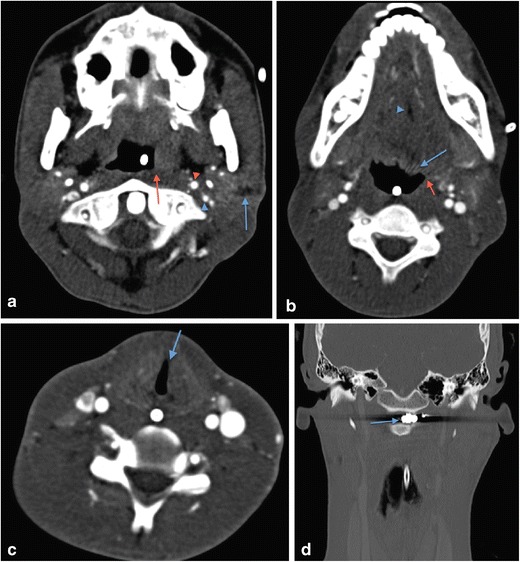
**a** Axial image of MDCTA of sharp (non-projectile) penetrating injury to the left zone 3. The implement bisected the left carotid sheath just below the skull base with the wound track (*blue arrow*) splaying the internal carotid artery (*red arrowhead*) from the internal jugular vein (*blue arrowhead*) but not causing any radiologically evident vascular injury. The patient exhibited clinical signs of Collet-Sicard syndrome—palsies of cranial nerves IX, X, XI and XII. The MDCTA demonstrates flattening of the left lateral pharyngeal recess (*red arrow*) compared with the contralateral side as a result of a combination of haematoma and superior pharyngeal constrictor paresis (the motor supply of the superior pharyngeal constrictor is through the motor component of the pharyngeal plexus derived from the cranial part of the accessory [XI] nerve). There is a nasogastric (NG) tube in situ. **b** Axial image of MDCTA of the same patient as in **a** showing evidence of left hypoglossal nerve palsy—there is flaccid prolapse of the tongue base (*blue arrow*) and deviation of the median fatty raphe to the ipsilateral side (*blue arrowhead*) and reduced calibre of the left glossopharyngeal sulcus (*red arrow*). The NG tube is noted in situ. **c** Axial image of MDCTA of same patient as in **a** and **b**. There is subtle medialisation of the left true vocal cord (*blue arrow*) consistent with a left vagus nerve palsy confirmed on flexible nasoendoscopy; this is one of the components of Collet-Sicard syndrome. The NG tube is evident in situ. **d** Coronal reformat of the same patient as in **a**, **b** and **c**. A metal fragment of the implement used to inflict the penetrating injury remains lodged in the left prevertebral soft tissues (*blue arrow*) having bisected the left carotid sheath and injured the adjacent cranial nerves

Vascular injuries are the most common injuries associated with penetrating neck trauma, estimated to occur in up to 40 % of patients [27] and with arterial injury representing between 15 % and 25 % [19, 24, 25, 31]. Of arterial injuries, 80 % involve the carotid vessels and up to 43 % involve the vertebral arteries [19, 33, 34]. Morbidity and mortality relate to complicating neurological deficits as a result of stroke (Fig. 3a) and not simply as a result of exsanguination. Physical findings caused by neurological deficits can include Horner’s syndrome or cranial nerve dysfunction (Fig. 5a, b) as well as overt anterior or posterior circulation stroke.

**Fig. 6:**
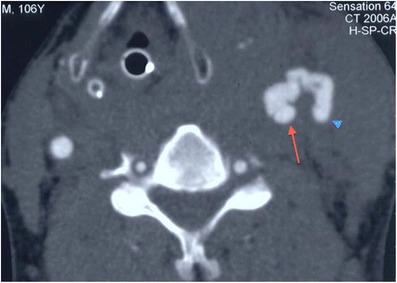
Axial image of MDCTA in an adult male patient who sustained a penetrating stab wound to the left zone 2 of the neck demonstrates a resultant traumatic left arteriovenous fistula between the left common carotid artery (*red arrow*) and adjacent internal jugular vein (*blue arrowhead*). It was subsequently closed through radiological intervention. Note the haematoma affecting the overlying left sternocleidomastoid muscle parenchyma and the large haematoma and oedema in the left parapharyngeal and extralaryngeal soft tissues causing mass effect and significant displacement of the laryngopharynx to the contralateral side. The patient is intubated. (The authors’ departmental protocol is to arbitrarily register the year of birth for any adult patient who is unconscious or intubated and ventilated, where details are unavailable, as 1900 in the acute admission situation—the patient’s age has therefore been “erroneously” calculated as 106 years by the scanner as shown in the top right-hand corner of this image)

**Fig. 7:**
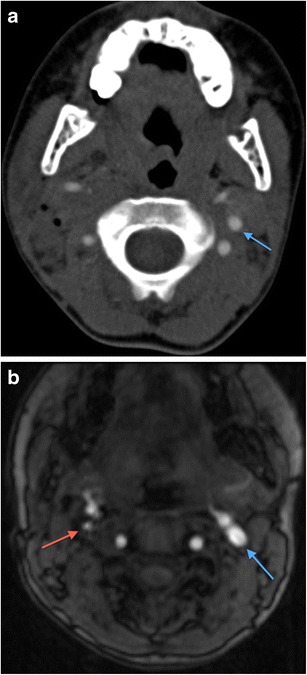
**a** Axial MDCTA image of a 9-year-old patient who sustained accidental penetrating injury by a wooden stick to zone 2 of the right neck. The stick was removed at the time of the injury by the patient’s friend but there is dissection occlusion of the right internal carotid artery; only the external carotid artery, one of its branches and the vertebral artery are visible on this side. The normal left internal carotid artery is annotated for comparison (*blue arrow*). The patient escaped any significant complicating cerebrovascular ischaemic event. **b** Axial source image of two-dimensional time of flight MR angiogram of the same patient as in **a**, performed 4 years later as part of follow-up assessment. The previously injured right internal carotid artery has re-canalised (in keeping with the natural history of dissection injury) but exhibits residual focal stenosis (*red arrow*) compared with the normal contralateral left internal carotid artery (*blue arrow*)

MDCTA accurately demonstrates the vascular injuries associated with projectile and non-projectile penetrating trauma of the neck. These include: pseudoaneurysm formation, arteriovenous fistula formation (Fig. 6), vessel transection, intimal injury with flap formation, dissection, active bleeding, and partial or complete occlusion of the carotid or vertebral arteries (Figs. 3b and 7a, b). The involvement of the internal jugular vein by penetrating injury is not infrequently identified on CTA and for this reason, in the authors’ experience, some early venous contamination on the MDCT acquisition is not always deleterious. Injury to venous structures should not be ignored: venous injuries occur in nearly 20 % of patients with penetrating trauma of the neck and are frequently missed at physical examination [35].

Studies comparing the accuracy of CTA (single-slice and multi-detector) with catheter angiography have prompted a shift of emergency vascular imaging assessment of appropriate penetrating neck injuries to MDCTA as the primary imaging modality and technique of choice [3, 18, 20–22, 27, 36]. The value of MDCTA in penetrating neck injury assessment of non-vascular injuries affecting the upper aerodigestive tract and osseous structures has also been consolidated [26].

The facility of catheter angiography in the management of penetrating vascular injuries of the neck should not be dismissed in its entirety. Although no longer the first-line diagnostic imaging modality of choice, catheter angiography may still be required as part of the management of certain arterial injuries identified on MDCTA in the haemodynamically stable patient amenable to therapeutic radiological endovascular intervention. In addition, particularly in the setting of projectile penetrating injury, where streak artefact from metallic fragments such as bullet fragments or shrapnel may, on occasion, hinder CT interpretation, catheter angiography may be indicated. An example of such a scenario might be where wound trajectory in relation to major vessels is of significant-enough concern, but CT interpretation is significantly hindered by streak artefact. Magnetic resonance (MR) imaging, and more specifically MR angiography, has no role in the imaging assessment of metallic projectile penetrating neck injury because of the presence of potentially ferromagnetic foreign body material. MR has only a very limited and selective role in the acute imaging assessment of non-projectile penetrating neck injury: where non-projectile penetrating injury to the cervical spine or craniocervical junction is suspected clinically, MR imaging will be essential [28], particularly as these patients frequently survive such injuries despite severe morbidity.

The use of ultrasound imaging in penetrating neck injury is very limited when compared with MDCTA, particularly given its inability to evaluate the upper aerodigestive tract and spine.

## Upper aerodigestive tract trauma

### Oesophageal and pharyngeal injury

**Fig. 8:**
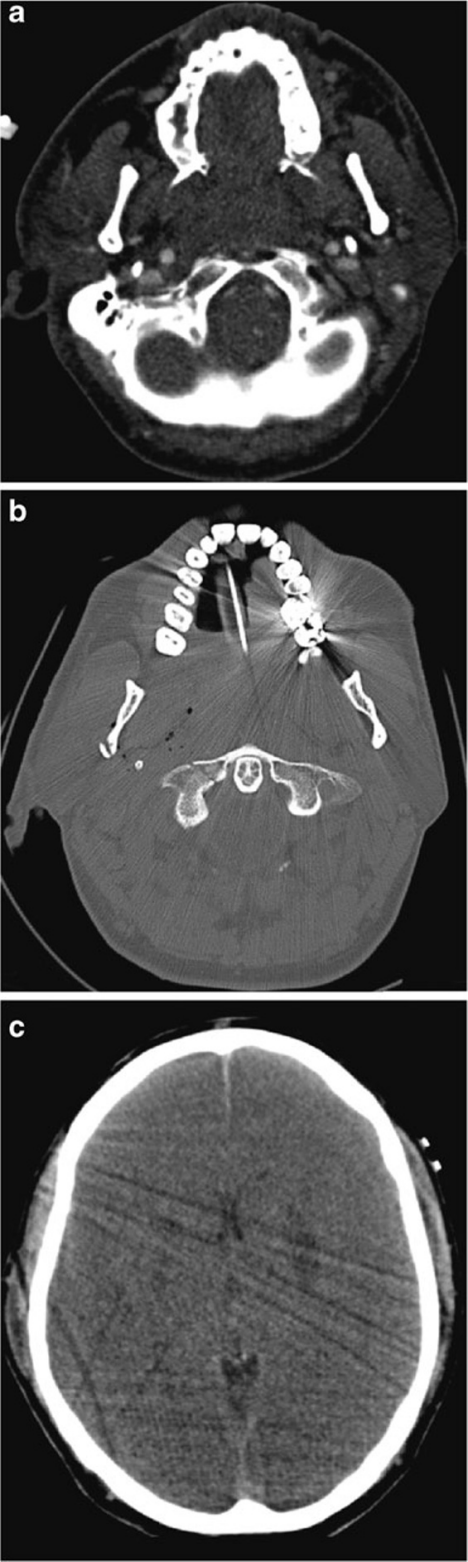
**a** Axial MDCTA image through the junction of the nasopharynx with the oropharynx (above the level of the endotracheal tube) in a 43-year-old man who sustained fatal perforating gunshot injury to the neck, which entered in the left zone 3 and exited through the right zone 2, having traversed the posterior oropharynx (image not shown). The pharyngeal airway at the level shown has been completely obliterated by haematoma and oedema. Pharyngeal projectile penetrating injuries, in the absence of major vascular injury, are frequently fatal due to airway compromise from haematoma, oedematous swelling and haemorrhagic debris. **b** Axial CT image of the same patient as in 8a on bone-window settings through the superior oropharynx (slightly inferior to the level in **a**). There is fracture disruption of the right lateral mass of the atlas (C1) at the junction with the anterior arch as well as fracture of the posterior aspect of the right ramus of the mandible cause by the bullet (which exited through zone 2 on this side). Soft tissue emphysema and swelling is evident in the right prevertebral, parapahryngeal and masticator spaces. The endotracheal tube is demonstrated. **c** Non-contrast CT brain of the same patient as in **a** and **b** performed 2 days later, demonstrating severe generalised hypoxic ischaemic encephalopathy with symmetrical subacute low attenuation change identified in the basal ganglia at this level as well as evidence of significant cerebral oedema and swelling. Brain stem death was subsequently confirmed and intensive care support withdrawn

**Fig. 9:**
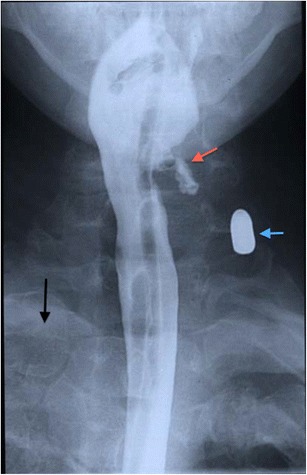
Contrast swallow assessment of a patient who sustained multiple penetrating gunshot injuries, including to the neck. A bullet is demonstrated in the soft tissues of the left zone 2 of the neck (*blue arrow*). A nasogastric tube was passed during the emergency trauma assessment and can be seen in situ. Haematoma is demonstrated in the left pyriform fossa with mucosal disruption and contrast leakage into the parapharyngeal tissues (*red arrow*). Note the opacification of the upper zone of the right hemithorax consistent with haemothorax (*black arrow*)

Injury of the pharynx and proximal (cervical) oesophagus is uncommon but should be considered where the trajectory of the penetrating injury comes into close proximity with either of these structures (Fig. 8). Delay in diagnosis is the most important contribution to the mortality associated with injuries to the oesophagus, estimated to be approximately 20 % [27, 37–39]. The proximity or otherwise of the wound trajectory can be assessed on MDCTA. Where clinical and radiological suspicion is high or frank injury to the oesophagus has been identified on MDCTA, contrast swallow assessment (if the patient is able to comply with the procedure), or endoscopy, or both will be indicated (Fig. 9). Although rigid endoscopy has, historically, always been performed where endoscopy has been required, some centres now practice flexible endoscopy [27, 40]. The main cause of mortality in patients sustaining oesophageal injury is mediastinitis and sepsis, hence the need for rapid early diagnosis of oesophageal injury and a high index of suspicion. Pharyngeal penetrating injury carries similar risks of delayed sepsis, descending retropharyngitis and resultant mediastinitis, but also the potential for immediate airway compromise; in the authors’ experience, this is particularly the case with gunshot injuries to this region. In a recent study, Ahmed et al. [41] reported contrast swallow studies to be less sensitive in detecting hypopharyngeal injury compared with oesophageal injury and have advocated the use of flexible nasoedoscopy in such patients. Bullet migration is a recognised phenomenon in gunshot penetrating neck injury and carries a risk of distal migration and aspiration of bullet fragments entering the upper aerodigestive tract and also cerebral embolisation from bullets entering the vascular tree or cardiac chambers at the entry wound site [28]. The identification and location of such fragments in relation to the upper aerodigestive tract and the possibility of such migration should be considered by the reporting radiologist [28, 42].

### Laryngotracheal injury

**Fig. 10:**
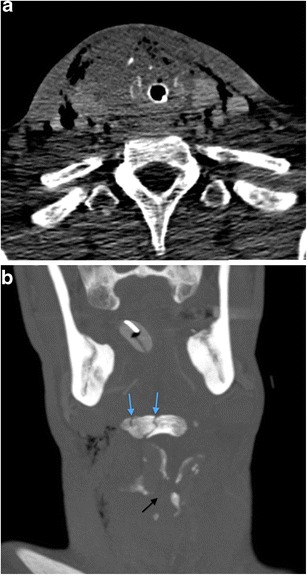
**a** Axial MDCTA image of a 38-year-old man who sustained a sharp penetrating injury to the midline zone 2 of the neck with a broken bottle (“glassing”). This particular mechanism is typically a combination of sharp penetrating and blunt laryngeal trauma. There is marked endolaryngeal, extralaryngeal and thyroid parenchymal haematoma, particularly on the right. Soft tissue emphysema can be seen outlining the pretracheal fascia which binds the thyroid gland. Further extensive soft tissue emphysema was demonstrated outlining the other deep cervical fascial layers, including the investing layer, the carotid sheath and the prevertebral/alar fascia layer (images not shown). Such extensive soft tissue emphysema should raise significant concern radiologically and clinically for penetration or perforation of the larynx. The patient has been successfully intubated. **b** Coronal-reformatted MDCTA image of the same patient as in **a**. Comminuted fracture disruption of the hyoid bone (*blue arrows*) and comminuted fracture-subluxation of the laryngeal cartilage (*black arrow*) are evident

**Fig. 11:**
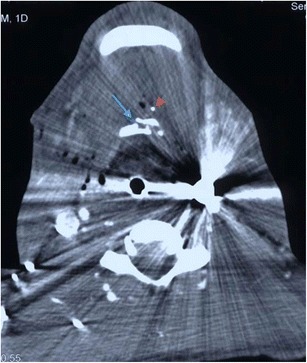
Axial MDCTA image through zone 2 in a victim of multiple gunshot injuries to the neck, chest and abdomen. The right superior zone 2 entry wound with associated soft tissue haematoma and oedema is evident. The bullet has traversed the supraglottis and come to lie in the left zone 2 in close proximity to the left internal carotid artery. The bullet has fragmented, with a resultant secondary projectile fragment (*red arrowhead*) causing fracture disruption of the hyoid bone (*blue arrow*). Despite the penetrating laryngeal injury, successful intubation has been achieved

Laryngotracheal penetrating injury is relatively uncommon, occurring in 1–7 % of patients and with cervical tracheal trauma prevailing [27, 39, 43–46]. Clinical examination may reveal significant subcutaneous emphysema; MDCTA exhibiting very extensive soft tissue emphysema that is far in excess of the wound appearances should alert the radiologist to the possibility of traumatic breach or disruption of the laryngotracheal airway (Figs. 10a, b and 11). However, less marked and relatively localised soft tissue emphysema can occur as a normal appearance in penetrating neck wounds not breaching the upper aerodigestive tract. Sometimes the site of laryngotracheal soft tissue breach or cartilaginous disruption may be easily identified on the CT assessment, but on other occasions, particularly in cases of laryngeal injury, extensive extralaryngeal and endolaryngeal soft tissue swelling and haematoma may conceal the site of direct injury [3, 45–47]; this can be particularly evident in projectile penetrating trauma due to gunshot injury where radiological interpretation may be further hindered by streak artefact. The potential for distal migration of endolaryngotracheal bullet fragments should be considered by the radiologist even in spite of the presence of tracheal intubation (whether this is in the form of endotracheal intubation, emergency tracheostomy or emergency cricothyroidotomy) [28, 42].

## Facial trauma

**Fig. 12:**
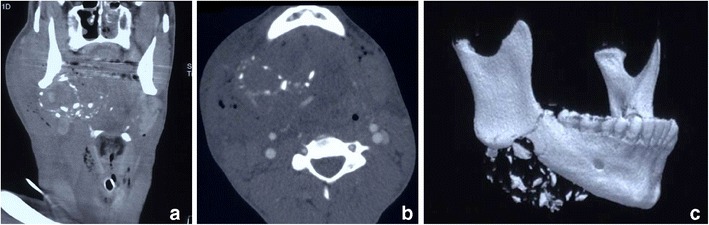
**a** Coronal reformatted MDCTA image of a 16-year-old man who sustained a gunshot injury to the right superior zone 2. There is a large haematoma in the right submandibular region, extending medially into the ipsilateral sublingual space and oral cavity, as well as laterally into the supero-lateral facial and buccal soft tissues. The comminuted fracture of the affected posterior aspect of the body of the right hemimandible has yielded numerous fracture fragments which have potentiated soft tissue damage. Because of marked haematoma and haemorrhagic debris affecting the oral cavity and oropharynx, emergency per-oral endotracheal intubation could not be performed and the patient has emergency cricothyroidotomy cannulation in situ. **b** Axial MDCTA image of the same patient as in **a**. The extensive soft tissue haematoma and oedematous swelling of the right oral cavity, oropharynx, sublingual space, submandibular space and facial region is evident as well as the in-driven comminuted bone fragments arising from the right hemimandible. Bilateral soft tissue emphysema is present. Note the absence of per-oral endotracheal intubation. **c** Three-dimensional VRT of the mandible demonstrating the relatively focal comminution of the right hemimandible caused by bullet impact and the resultant dispersal of in-driven bone fracture fragments. Three-dimensional volume-rendered images can be particularly helpful to the maxillofacial surgeon managing such injuries and also in assisting criminal police investigations and court presentations

**Fig. 13:**
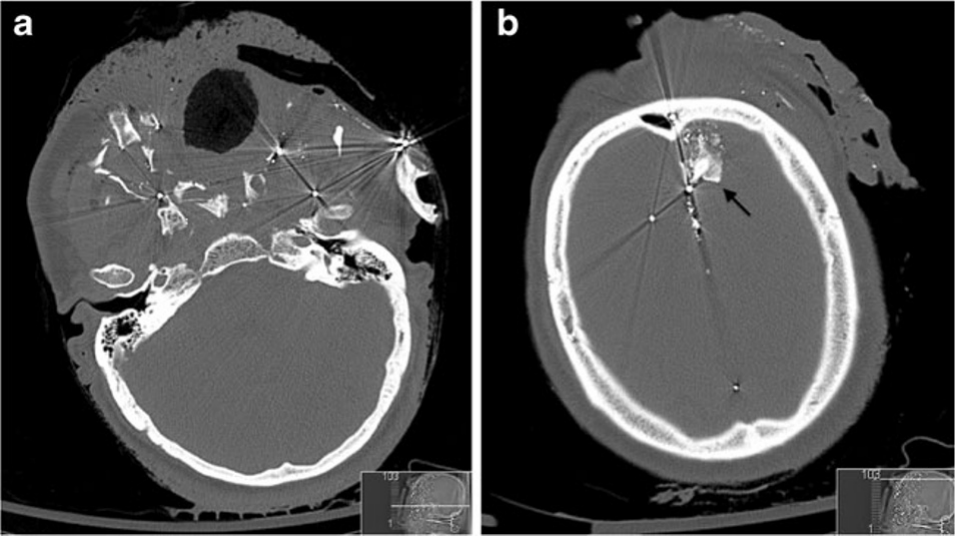
**a** Axial non-contrast CT face image on bone-window setting of a 51-year-old man with self-inflicted shotgun injury to the face. The high mortality of self-inflicted shotgun injuries to the face relates to the high tissue-destructive energy of a shotgun fired at close range to the target (compared with long range) as well as the “billiard-effect” created by the shotgun pellets. In this case, the marked avulsion of the facial soft tissues is evident as well as the marked bone disruption and fragmentation of the facial and mandibular skeleton. **b** Axial non-contrast CT image through the brain on bone-window settings of the same patient as in **a**. An in-driven canine tooth and attached alveolar bone fragment (*black arrow*) has penetrated the left frontal lobe, in addition to some of the shotgun pellets. The in-driven fragments of facial skeleton in orbito-facial projectile penetrating injury may become significant secondary damaging projectiles, particularly to the brain

**Fig. 14:**
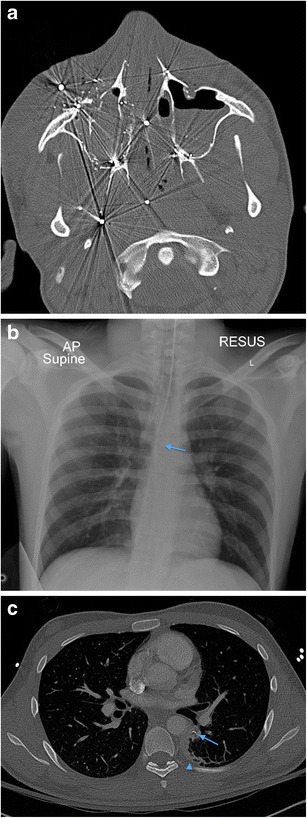
**a** Axial non-contrast CT face image in a 19-year-old male victim of a shotgun injury to the orbito-facial region. Multiple shotgun pellets are identified in the malar soft tissues, nasal fossae, maxillary sinuses, nasopharynx and masticator space, particularly on the right at this axial level. Fracture fragmentation of the right maxillary sinus, malar and retroantral haematoma and antral haemorrhagic fluid are present. The involvement of the nasal, nasopharyngeal and oral (the latter not shown) airway raises potential for distal migration of pellets (as well as of in-driven tissue fragments) through the upper aerodigestive tract into the lower respiratory tract and gastrointestinal tract. **b** Plain chest radiograph in the same patient as in 13a performed during trauma survey on admission demonstrates distal migration of a shotgun pellet (*blue arrow*) into the mid-oesophagus (confirmed on CT chest assessment [not shown]). The patient is intubated. **c** Axial image of a contrast-enhanced CT thorax of the same patient as in **a** and **b**, performed 12 h later also confirms aspiration of a shotgun pellet (*blue arrow*) associated with distal subsegmental left lower lobe atelectasis (*blue arrowhead*). There had also been further distal intra-oesophageal migration of the mid-oesophageal shotgun pellet (image not shown)

Penetrating trauma to the face may carry significant morbidity and mortality, not least because of the associated intracranial component of injury which may accompany such trauma. (This component will not be discussed further in this review). It can result from projectile penetrating injury, most typically gunshot injuries (and less typically blast injuries), or from non-projectile mechanisms. Direct gunshot wounds to the face encountered in the civilian setting are not uncommonly self-inflicted, in which case the weapon of choice tends to be the shotgun. The imaging modality of choice remains non-contrast CT of the facial bones and brain. In certain situations, MDCTA may also be appropriate. The reporting radiologist should be aware of the substantial secondary tissue damage caused by in-driven fragments of bone and teeth, which themselves become secondary projectiles (Figs. 12a–c and 13a, b). Shotgun pellets at short range cause significant propagation of tissue damage as a result of the “billiard-effect” of the individual pellets [28, 29, 42, 48–51]. In addition, the potential for shotgun pellets, bullet fragments or native organic tissue such as fragmented bone and teeth to enter the oral cavity, migrate and be aspirated, should also be considered by the radiologist when assessing the emergency imaging (Fig. 14a–c). There has been no documented evidence of lead poisoning caused by swallowed lead-containing bullets or shotgun pellets.

**Fig. 15:**
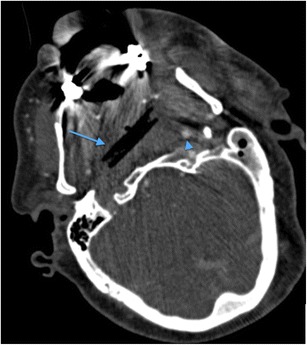
Axial MDCTA image through the infratemporal fossa of a 59-year-old man who fell on to a garden bamboo stick (*thin blue arrow*) which penetrated the left buccal region of the facial soft tissues, lodging and breaking-off in the retropharyngeal region of the nasopharynx and causing dissection occlusion of the right internal carotid artery (note the normal contralateral left internal carotid artery [*blue arrowhead*]). The geometric margin of the air-containing abnormality highlights the radiologic features consistent with an embedded foreign body fragment of organic material (wood). The patient subsequently developed a near-total thromboembolic right middle cerebral artery territory infarct (imaging not shown)

Non-projectile penetrating facial trauma most often results from injury by sharp implements such as knives, glass or screwdrivers, as well as accidental sporting or occupational impalement injuries caused by metal railings, garden materials such as sharp wooden poles and fencing or tree branches. The ability of such objects to penetrate deeply to the parapharyngeal and retropharyngeal compartments at the skull base where vital structures such as the internal carotid arteries course and may be at risk, should be considered. The possibility of more local superficial soft tissue damage such as parotid parenchymal or parotid duct injury should also be considered. Damaged external carotid artery branches can be a source of catastrophic haemorrhage, which may warrant emergency endovascular treatment. MDCTA may be of significant value in such circumstances. Organic material such as impaled wood fragments can be somewhat elusive on CT assessment unless appropriate window settings are employed, depending on the amount of air and fluid within the interstices of the wood [52–54]. Impaled wood fragments may appear as “air” on CT and MRI assessment, but the radiologist should be alert to the possibility of an embedded fragment of wood if this “air” exhibits a geometric margin (Fig. 15).

## Conclusion

The challenges facing the radiologist in imaging assessment of penetrating trauma of the neck and face are numerous and impact significantly on the effective management of these patients in the acute setting. Knowledge of the current recommended imaging protocols (primarily MDCTA) and relevant radiological appearances in this complex anatomical area can affect outcome significantly.
